# Therapeutic Affordances of Online Support Group Use in Women With Endometriosis

**DOI:** 10.2196/jmir.5548

**Published:** 2016-05-09

**Authors:** Amie Shoebotham, Neil S Coulson

**Affiliations:** ^1^ Division of Rehabilitation & Ageing School of Medicine University of Nottingham Nottingham United Kingdom

**Keywords:** endometriosis, social network, social support, qualitative research

## Abstract

**Background:**

The Internet has provided women living with endometriosis new opportunities to seek support online. Online support groups may provide a range of therapeutic affordances that may benefit these women.

**Objective:**

To examine the presence of therapeutic affordances as perceived by women who use endometriosis online support groups.

**Methods:**

Sixty-nine women (aged 19-50 years, mean 34.2 years; 65.2% (45/69) United Kingdom, 21.7% (15/69) United States) participated in a Web-based interview exploring online support group use. Participants had been using online support groups for an average of 2 years and 4 months (range = 1 month to 14 years, 9 months). Responses were analyzed using inductive thematic analysis.

**Results:**

The analysis revealed 4 therapeutic affordances related to online support group use: (1) “connection,” that is, the ability to connect in order to support each other, exchange advice, and to try to overcome feelings of loneliness; (2) “exploration,” that is, the ability to look for information, learn, and bolster their knowledge; (3) “narration,” that is, the ability to share their experiences, as well as read about the experiences of others; and (4) “self-presentation,” that is, the ability to manage how they present themselves online. The associated outcomes of use were predominantly positive, such as reassurance and improved coping. However, a number of negative aspects were revealed including the following: concerns about the accuracy of information, arguments between members, overreliance on the group, becoming upset by negative experiences or good news items, and confidentiality of personal information.

**Conclusions:**

Our findings support the previously proposed SCENA (Self-presentation, Connection, Exploration, Narration, and Adaptation) model and reveal a range of positive aspects that may benefit members, particularly in relation to reassurance and coping. However, negative aspects need to be addressed to maximize the potential benefit of support groups. Some of these can be addressed relatively easily through making privacy policies clearer, including health professionals to moderate content, and structuring forums to encourage the sharing of positive stories.

## Introduction

Endometriosis is a chronic condition that affects women of reproductive age. It is defined as the presence of endometrial tissue outside the uterine cavity, which induces a local inflammatory response [1]. Common symptoms include chronic pelvic pain, dysmenorrhea, fatigue, heavy menstrual bleeding, and dyspareunia. Estimates of the prevalence of the condition range between 2% and 17% of the female population [2]; however, the prevalence can increase to up to 47% in women with fertility issues [3]. Women typically experience a delay before diagnosis [4-6] with long-term care focused on symptom management [7,8]. Psychosocial effects are also common, including high levels of depression and anxiety [9-11], emotional distress [12,13], and poorer quality of life [9,14]. Endometriosis can negatively affect social life, work, and daily activities [15-17] as well as education, finances, life opportunities [18], personal relationship quality [8], and physical intimacy [19].

Women living with endometriosis typically need considerable support to meet the various challenges associated with the condition [20], and access to it may yield benefits through a reduction in illness-related stress [21] and improvements in well-being [22]. However, women with endometriosis often describe a lack of support [23,24], encounter feelings of social isolation [13], and find it difficult to disclose their condition and symptoms to others because of embarrassment or a fear of not being believed [16]. It can be difficult for these women to find an understanding and knowledgeable source of support, especially as the support may be needed for many years [20]. Finding other women with endometriosis, who understand the constant struggles, is therefore potentially a valuable source of support for them [16,20]. However, women often lack contacts with other women with the condition [23], or they may be unable to attend local support groups, should any exist, because of pain restrictions. The Internet therefore becomes a key resource when seeking the support of other women living with endometriosis [16,20].

Over recent years, the popularity of using the Internet for information and support has been growing; in 2013, 43% of adults in the United Kingdom [25] and 72% in the United States [26] accessed health information online. Furthermore, up to a quarter of Internet users have gone online to read about other people’s experience of a health-related problem and 16% to find others with the same problem [26]. The popularity of using the Internet for such purposes may be explained by its flexibility; it does not have the same restrictions as face-to-face support groups with regard to set times or locations [27]. The lack of face-to-face contact can also help users feel less inhibited and talk openly about embarrassing topics [28] or stigmatized health conditions [29].

Previous research has suggested that women with endometriosis do find the Internet to be a valuable source of information regarding both the condition and its medical management [30,31]. To date, however, there is lack of research into the use of online support groups for endometriosis. In contrast, research exploring the role of online support groups across other long-term conditions has been extensive and has found their use to have benefits including the provision of emotional support [32], instilling hope [33], fostering empowerment [32,34], and reducing feelings of isolation [35].

However, although such benefits are common, there can also be negative aspects of online support group use. Research has indicated that their use can lead to distress, particularly when reading about others’ negative experiences [35]. Users can also become obsessed with the groups [35] or display disinhibition due to the anonymous interface of the groups [28]. Furthermore, there is a risk that the medical information that is shared online is potentially inaccurate [36].

In order to understand how the use of online support groups might influence the outcomes of those living with endometriosis, this study considered the therapeutic affordances of online support group use. The origins of affordance theory stem from perceptual and cognitive psychology and are based on how individuals perceive the objects in their environment, pertaining to both what the object is and what potential uses it affords [37]. The properties of an object will therefore contribute to its perceived affordance. Furthermore, the differing experiences, beliefs, and goals of an individual will lead to different perceived affordances, and so affordances are unique to each individual [38]. The emphasis in the theory is on the interaction between the user and the object and its resulting outcomes.

Therapeutic affordances can therefore be seen as the “actionable possibilities” of the object as determined by the user [39], and the object in this instance is online support groups. Studying therapeutic affordances allows us to look at both the uses and effects of online support groups. This research will therefore provide further insight into the use of online support groups than previous research, as it will examine the underlying mechanisms that drive the observed effects and outcomes of support group use [39].

Previous research on therapeutic affordances within a health context is lacking. However, Merolli et al [39] examined the therapeutic affordances of social media use in people with chronic pain. They identified five therapeutic affordances of social media: self-presentation, connection, exploration, narration, and adaptation. The results highlight both the “actionable possibilities” of social media use, that is, connection, and the therapeutic value of such, that is, not feeling alone. Their work provides a good point of comparison for our study; however, there are important differences such as the health condition being studied and the different “object.” Online support groups have different properties than social media and therefore may enable different affordances. Specifically, online support groups tend to use an asynchronous discussion forum format, through which individual group members can post messages to start new conversations, or reply to messages within existing conversations, thereby creating a tree-like structure of discussion threads.

The aim of this study therefore was to explore the therapeutic affordances of online support group use in women with endometriosis and the resulting outcomes. The results of this research will yield a richer understanding of both the therapeutic benefits of online support groups and the needs of women living with endometriosis.

## Methods

### Procedure

This research involved the recruitment of women (aged 16 years or older) who use online support groups for endometriosis. They were recruited through messages posted onto the boards of such support groups, during a 4-week recruitment period from June 16 to July 13, 2015. In order to identify potential recruitment channels, searches were conducted on Google, using terms such as “online support group endometriosis” and “endometriosis support group,” as well as similar searches on Facebook. The groups that appeared to be the most recently active (some had been dormant for a number of months) were selected as potential recruitment channels.

Initial contact was made with the board moderators to seek permission to recruit their group members via a message posted onto the boards of the groups. It was made clear to the moderators that the research focused on women’s experiences of using such groups, and they were given the opportunity to review the message before posting. Once permission was granted, the recruitment message was posted, which explained the aims of the study and invited those interested to click on a link to the survey, hosted by Bristol Online Surveys. There was a degree of variation in the final boards used for recruitment, having 1 general endometriosis online support group, 1 board hosted by an online fertility support group, and 1 group hosted by Facebook.

When directed to the survey page, participants were provided with additional information about the study, and they were then required to complete a consent form before beginning the survey. Participants first completed a series of short answer questions relating to their background (date of birth, country of residence, diagnostic status, and so on) and use of online support groups (frequency, period of use). Next, they were invited to respond to a set of open-ended questions that explored their motives and experiences of using online support groups and whether their use has any effect on how they cope with or manage the condition.

### Data analysis

In order to provide a rich account of the data, the responses to the open-ended questions were qualitatively analyzed using deductive-inductive semantic thematic analysis [40]. This analysis approach allows for the identification of common themes across the dataset and therefore closely reflects the data and the language used by participants. This is particularly important given the aims of this study.

The analysis was carried out by the first author (AS) according to the guidelines proposed by Braun and Clarke [40], and QSR’s NVivo 10 software was used to maintain an audit trail. First, to become familiarized with the data, the transcripts were read and reread several times and initial ideas about the data were noted down. Next, interesting features of the data were coded, keeping codes close to the language used by participants where possible. Codes were then organized into meaningful groups to form potential themes. Codes that appeared consistently throughout the data were automatically considered as a potential inductive theme. Data relevant to each potential theme were gathered, and then themes were reviewed, refined, and given a clear definition and name. Reviewing the common language used in these themes allowed them to be organized into the final therapeutic affordances. To reduce bias and check whether the themes reflected the data, an independent researcher read through some of the transcripts and agreement was reached on the final themes.

### Ethical Considerations

Before data collection, the research was reviewed and approved by the University of Nottingham (United Kingdom) institutional ethics review committee. In order to maintain ethical requirements, participants were provided with full study information that informed them of their rights as a research participant, procedures of withdrawal, and full contact details of the researchers. Before beginning the survey, participants were required to complete a Web-based consent form indicating that they understood such rights. They were each asked to provide a unique password to allow for their data to be withdrawn if they so wished after survey completion and were given a time frame within which they could withdraw. They were informed that, to maintain privacy and confidentiality, no personally identifiable information would be reported in the results. Furthermore, they were able to indicate during consent if they were happy for their responses to be quoted in research reports (3 participants chose not to have their responses quoted). In order to meet ethical requirements, quotes provided in the report only identify participants by their responder number (ie, Respondent 17).

## Results

In total, 69 participants completed the Web-based survey. The respondents were aged between 19 and 50 years, with a mean age of 34.2 years, and the majority were residents of the United Kingdom (65.2% 45/69) or the United States (21.7% 15/69). Of the respondents, 66 (95.7%) had received a confirmed diagnosis of endometriosis, which was made between 1 month and 20 years before survey completion (mean time since diagnosis = 4 years, 1 month). Participants had been using online support groups for endometriosis for between 1 month and 14 years, 9 months (mean use period = 2 years, 4 months), and most participants (59.4% 41/69) accessed the groups more than 5 days a week.

Following deductive-inductive thematic analysis, the therapeutic affordances that were present in the data were “connection,” “exploration,” “narration,” and “self-presentation.” Quotes were largely positive; however, participants did refer to some negative aspects afforded by online support groups. Table 1 describes the inductive themes that emerged from the data analysis within each affordance and the key language used by participants.

**Table 1:** Final therapeutic affordances, inductive themes, and descriptive language.

Therapeutic affordance	Themes	Language
Connection	Contact with others with endometriosis Support Exchanging advice Forming relationships Mitigating isolation Freedom of access	Talk Advice Alone Support Friends Community Understanding
Exploration	Information seeking Credibility Learning/knowledge Empowerment	Find Learn Discover Look Know
Narration	Sharing experience Reassurance Disheartening	Stories Share Hear Experiences Competition Negativity
Self-presentation	Privacy Disclosure	Confidential Private Embarrassing

### Connection

The most frequently cited affordance of online support groups was the ability to connect with others, and participants used this connection to support each other, exchange advice, and to try to overcome feelings of loneliness. They benefited from the freedom to connect with a large number of individuals and at all hours of the day or night.

Participants frequently commented on their ability to connect with other women who have endometriosis through the online support groups. They demonstrated a sense of relief at finding those who “understand” what they are going though: “...to be able to connect with others suffering the same just gives you some piece of mind” [Respondent 28] and “...having a place to speak freely with people who completely understand & offer love, encouragement & advice is a great help at times of need” [Respondent 11]. Finding an understanding support network had also significantly helped women to cope with the condition: “After years of struggling with GP's finding other people who understood played a huge part in making me feel normal again” [Respondent 25] and “I think one of the reasons I haven't had a nervous break down or gotten depressed is because of the groups. I have people who understand what I'm going through and can give me advice” [Respondent 10].

Participants spoke positively of the support they were able to offer each other through online support groups: “The support is great” [Respondent 29] and “[I] have found the support online invaluable” [Respondent 4]. In some instances, this support had a marked effect on women’s lives, enabling improved coping and confidence in seeking better treatments: “...talking through some of the darker times with women who know what I’m going through has been hugely helpful and I feel I am coping better than I was before” [Respondent 16], “I also received support from lots of ladies when I didn’t know how to make myself heard...” [Respondent 32], and “I am also going to ask to be referred to a specialist because of support from others on this forum” [Respondent 5].

Receiving support was described by many as a key motivation for online support group use. However, some women also talked favorably about providing support to others, finding the process to be rewarding, and even of psychological benefit: “I feel I can provide some help & support to the ladies because I have a lot of experience...” [Respondent 11] and “...the rewarding feeling following directly supporting others has improved my mental wellbeing...” [Respondent 16].

A large number of participants spoke about how online support groups had allowed them to share advice, either on general matters, or those related to treatments or self-management behaviors. Participants often used words such as “appreciate” or “valuable” when talking about advice received from others: “The advice and support received is invaluable...” [Respondent 25], “I also really appreciate the advice and support these groups offer” [Respondent 27], and “I have had valuable advice on symptom relief, side effects of treatments, post op care etc etc” [Respondent 51]. Women also found it helpful to provide advice to others, experiencing a positive feeling from doing so: “...I feel like it is helpful to me to be able to help other women, with comments and advice” [Respondent 13].

Advice in general was gratefully received, although some participants expressed caution at the medical advice shared through these platforms: “...I’m not going to take medical advice through Facebook” [Respondent 42]. Some indicated that although they do read the advice, they would usually ultimately check it with their medical professionals and follow their advice: “I feel that the online group helps answer any questions...but I have always gone ahead with what I thought or the medical staff say” [Respondent 33] and “I do listen to the advice but in the end I usually go with what my consultant suggests even if it's not what I want” [Respondent 67].

The most frequently occurring theme within “connection” was that of mitigating isolation; the online support groups had allowed participants not to feel alone. The word “alone” was mentioned by several respondents, and many talked about the isolating nature of endometriosis: “I feel less alone in the groups- I know I'm not the only one experiencing this” [Respondent 22], “Also knowing you are not alone in your struggle, as endometriosis can be very isolating...” [Respondent 9], and “I was feeling so alone and very down before I discovered there was others just like me who understood what a struggle constant pain could be” [Respondent 25].

Having found others in a similar position to themselves, women were able to feel acceptance of their condition: “Now I know that there are other women suffering I can accept that this is actually happening to me!” [Respondent 22] and “...knowing that what I'm feeling is real...that I'm not alone” [Respondent 23]. They were also able to feel reassured about any unusual symptoms experienced: “There are symptoms I've experienced that I thought was unique to me only to find that many others have the same issues. It was through the online support groups that I found I wasn't alone” [Respondent 60].

Connections made through online support groups could be very strong, with some talking of the relationships they had formed with other people. They often used words such as “friends” and “community” when talking about their connections with others: “I've made quite a few friends who I speak with regularly” [Respondent 22], “I've made good friends” [Respondent 20], and “...I have increasingly used the forum to seek community...” [Respondent 8]. One woman particularly emphasized the role of these online support groups in enabling connections she could not have found elsewhere: “I find a sense of community there that I can’t find in the physical world” [Respondent 8].

However, connecting to others through the support groups did not always foster positive outcomes. Participants alluded to problems in communication that they had witnessed between other members, such as arguments: “There are sometimes arguments about what is the best treatment” [Respondent 22] and “I have seen many posts get out of order & things can escalate quickly...” [Respondent 11]. This was also reiterated by a member of the administration team for one support group: “As an admin on [name of OSG], I do sometimes have to deal with arguments and the usual sort of online drama...” [Respondent 50]. Although such incidents caused frustration, they were only mentioned by a minority of participants. One participant even found the positive side to it, citing it as a source of entertainment: “I visit the group now far more due to fear of missing a scandal! It's a hive of bitchiness - better than a soap opera” [Respondent 18].

The final theme relating to “connection” was how online support groups allowed participants the freedom to access people. This could be access to a large number of people: “In the groups I could ask 100s /1000s of people questions...” [Respondent 22]; or access at any time of the day or night: “The main benefits of being in an online support group is that you have access 24/7” [Respondent 1]. Access to such high volumes of people meant that online support groups enabled women to get help and support quite quickly if they needed it: “Any time I have a question I can post and get reassurance and answers rather quickly” [Respondent 22]. This could be particularly comforting at times when participants were suffering physically or psychologically; “...I have had three laparoscopies in the past and to know others are there whenever in need is lovely” [Respondent 29] and “Knowing that there is always someone there to listen when things get bad or I am feeling down” [Respondent 67].

A small number of women described potential pitfalls arising from this freedom of access. Having constant access to such platforms could cause preoccupation: “Possibly a bit pre occupied with the group” [Respondent 41] and “My life revolves round Facebook etc because I can do so little” [Respondent 18]. Furthermore, one woman felt that access to so many people actually negated the support she was seeking: “No one responds to my posts. There are too many posts/people and not enough support to go around” [Respondent 24].

### Exploration

The second largest affordance of online support groups for those with endometriosis was “exploration.” Women were able to look for information, learn, and bolster their knowledge, and in doing so appeared to feel more empowered.

Participants frequently described how their use of online support groups had enabled them to seek out information, from general information about the condition to more specific information on treatments, research, and specialists. Many commented on how they were able to find information that had not been provided by medical professionals: “Additional information that may not have been given by my own consultant” [Respondent 44] and “I do not think I would have obtained the high quality of this information from the medical community directly” [Respondent 37]. Again, positive words were used when describing the information gathered through online support groups: “...obtaining invaluable information on the condition...” [Respondent 14] and “...and overall wealth of information has been immensely helpful” [Respondent 38].

Although most participants valued the information found through this platform, some expressed concerns about the quality of information being given on such sites: “However it was / is also a minefield of myths and misinformation...” [Respondent 61] and “I feel like someone a bit younger might fall pray to some of the misinformation some women provide” [Respondent 2]. In a similar vein, some questioned the credibility of the sources of information and were concerned about people self-appointing themselves as experts: “There are some who think they are experts but really are not” [Respondent 51]. Participants generally felt the information was credible if it came from a medical expert: “I tend to only really listen to the advice from the specialists who post and answer questions, regarding current research” [Respondent 55]. Alternatively, a number of women stated that they will do further research on any information obtained: “I conduct my own web based research in order to validate information I have obtained from online support groups” [Respondent 37].

A common theme talked about by participants was how online support groups had facilitated their learning and knowledge. This learning encompassed a wide range of factors, as it could be about the disease itself, the symptoms, treatment options, or self-management techniques: “In the forums I've learned so much about what the disease is, the proper surgery (excision vs ablation), the best doctors, and how to deal with the day to day misery of this disease” [Respondent 62] and “...I've learned about books to read, and diet, and supplements, and various other ways to deal with the symptoms” [Respondent 20]. Again, participants alluded to how the learning facilitated by these groups went above and beyond what they could have received from their doctors: “The information received from the medical community doesn’t even come close to the details of what I have learned in online Support groups” [Respondent 37].

This new knowledge received through the online support groups facilitated improvements in the self-management of the condition: “It has improved my health literacy around managing my own condition, which I am really grateful for” [Respondent 8] and “I know about staying ahead of the pain now. In times where I have previously gone to hospital with extreme pain I have piggy-backed painkillers as suggested in forums and been able to manage things at home” [Respondent 19]. It also helped to foster a sense of reassurance for the women, that what they have been experiencing is normal: “I also understand a lot more so I can rationalise what is happening to me” [Respondent 22] and “It has helped me understand why I have continued pain, despite multiple surgeries, and what I need to do to hopefully help revolve [*sic*] or at least reduce it” [Respondent 50].

A particularly pertinent outcome of the learning and knowledge facilitated by online support groups was a sense of empowerment. This was mentioned by many participants: “Knowing more about the condition from the group has made me more empowered” [Respondent 19] and “I feel a lot more empowered now that I have a reasonable understanding of the disease, this allows me to feel more in control and be better informed in the decisions I make about treatment” [Respondent 61]. In fact, many participants talked about how they now felt in control or more knowledgeable about their treatment options. As a consequence, they were able to attend medical appointments feeling more prepared and confident: “...the information obtained from online support groups helps me to be more prepared when speaking with medical practitioners...” [Respondent 37] and “I feel more confident about seeing medical professionals and getting a good standard of care as the support groups have provided me with sources of information that I have used to educate myself” [Respondent 19].

### Narration

Although slightly modest in size compared with the previous two affordances, participants did frequently comment on their use of online support groups to share their experiences, as well as hear the experiences of others. Through such narration, and that of others, participants were provided with a sense of reassurance, although at times it could also cause feelings of distress and disheartenment.

The majority of the “narration” quotes related to accessing the experiences of others, but a few did talk about how they had shared their own experiences with the condition. In doing so, participants described positive feelings and reassurance: “My mental health has improved by being able to share my experience...” [Respondent 16] and “I had a few really painful examinations I shared this with the group and found support and was also told this was not uncommon with woman with endo...” [Respondent 68]. Some also mentioned the benefits of sharing experiences as a two-way process: “I also benefit from the fact that we are able to discuss and share our experiences and outcomes with specialists...” [Respondent 50] and “It is nice to share stories and hear from others about their journey with endometriosis” [Respondent 3].

The responses that related to accessing the experiences of others were relatively evenly spread with regard to whether they fostered positive or negative outcomes. On a positive note, participants talked about how hearing those experiences have helped them: “Seeing other people's stories, be it success or negative stories as you can learn from both and gain some insight...” [Respondent 62] and “The personal experiences the other women have had and their outcomes of different treatments. Helps me to decide what to do next” [Respondent 43].

The experiences of others provided participants with feelings of reassurance about what they themselves had been experiencing. For example, one woman stated, “I feel reassured that many women with endo have similar frightening symptoms” [Respondent 7]. Another found that it helped to validate her own experience: “Hearing the testimonies of women living all over the world and knowing that what I'm feeling is real...” [Respondent 23]. Others simply found reassurance when reading others’ experiences by realizing that things could be worse, for example: “it's made me realise how lucky I am that I manage very well day to day compared to a lot” [Respondent 4].

However, many women also found that accessing others’ experiences could be disheartening. Some commented on the tendency for others to only share sad or negative stories: “There are so rarely any positive posts. People only seem to feel the need to post when they have something bad to report” [Respondent 18] and “The problem is that everyone talks about problems, very bad stories that make you feel more depressed, you never read happy things or happy stories!” [Respondent 42]. Hearing such negativity from others could be quite distressing: “Have felt disheartened and even a little depressed when reading some women's experiences...” [Respondent 49] and “...people repeating on multiple posts that it destroyed their lives to not have kids. As someone who has been diagnosed since 14 and is only 22 now it can be very overwhelming...” [Respondent 65].

At times participants described the propensity for some women’s narratives to be almost competitive in nature, which was seen as counterproductive, as it prevented them from wanting to share anything themselves: “I rarely comment in these groups now because it's like a competition. My endometriosis is worse than your endometriosis. I need to take more pills than you. My gynaecologist says mine is the worst case he's seen etc.” [Respondent 18]. One woman also talked about the opposite, how other women could be patronizing about the severity of others’ symptoms if they had not had them as badly themselves. The negative outcomes she felt were quite extreme: “There are also other ladies who tend to be a bit patronising and don't accept that endo can be different for everyone...This can make me feel quite insecure and pathetic when I am off work for the 6th time this year...” [Respondent 32].

Finally, although it was mentioned by many that there was a tendency for women to post negative stories, many participants reported that when positive stories were shared, such as a new pregnancy, it could cause a lot of distress in a group of people who frequently suffer from fertility issues. Although none of the women themselves said they felt this way, they often reported seeing other people getting upset about it, and sometimes even having arguments: “I have seen other members argue with each other about posting sensitive information, such as posting pregnancy announcements without ‘trigger warnings’...” [Respondent 37] and “There are those that, like myself, are infertile because of Endo. They cannot stand to see women make announcements that after having surgery or some form of treatment they are now pregnant...they will get upset and type angry rants and basically shoot down another EndoSister that is having a good experience right now” [Respondent 60].

Therefore, it would appear that rather than the potential positive outcomes of sharing such success stories such as feelings of hope, they may in fact exacerbate the distress felt by some women about their condition.

### Self-Presentation

This affordance relates to how the users present themselves to others via online support groups. Although only a small number of participants alluded to this affordance, those that did placed great emphasis on its importance. To some, the confidential nature, or “privacy,” of the support groups was beneficial: “Knowing i can discuss something, in private, amongst fellow sufferers” [Respondent 58] and “I knew it was confidential because it was a closed group. Only people within the group could see- not all my facebook friends and family!” [Respondent 22]. Having this private, anonymous platform enabled some to disclose matters they might not usually, or allowed them to “preserve” their offline relationships: “It helps me deal with it ‘privately’ because I don't have to ‘bother’ the people I am close with and spend every day with about my health concerns. I just ask my endo sisters” [Respondent 13] and “Having an outlet for sharing personal and embarrassing symptoms...” [Respondent 16].

Conversely, some worried about how confidential the platform really was, stating fears over whether other online friends were able to see what they were posting: “One concern that I have is the fact that I am posting very sensitive medical and personal information about my symptoms onto Facebook, and I do not feel that my personal information is secure on this platform despite the fact that these online support groups are on ‘secret’ pages” [Respondent 37]. “The only problem I have is that not all the support groups are ‘secret’ or private. Though sometimes I like to share the details of this disease with others who experience the same, I don't particularly want everyone of my Facebook friends to know about all my period pains or symptoms such as infertility” [Respondent 39].

This issue appeared to be mainly apparent in those who were recruited from the online support group hosted by Facebook, on which users are not anonymous, and usually go by their own name rather than a pseudonym. However, their language used indicates it as an important factor in their use of online support groups.

## Discussion

The aim of this research was to explore the therapeutic affordances of using online support groups for endometriosis. The results revealed 4 therapeutic affordances in this population: “connection,” “exploration,” “narration,” and “self-presentation.” In comparison with the results of previous research of online support groups, this study has uncovered similar therapeutic effects, including emotional support, reduced isolation, improved coping, reassurance, knowledge, and empowerment [32,34,35]. However, this research has also enabled a more in-depth exploration of the underlying mechanisms that generate the observed therapeutic outcomes, which suggests that the study of therapeutic affordances is a valuable way to research online support groups. Furthermore, the positive outcomes observed from online support group use have provided an indication of the potential influence that social support can have on those who are managing endometriosis.

The resulting therapeutic affordances are comparable to those found by Merolli et al [39] in their research on social media use in people with chronic pain. They too found the same 4 therapeutic affordances; however, their research also uncovered a fifth affordance, “adaptation.” This refers to the way that social media allows users to adapt their self-management needs in relation to their health status or illness flare-ups. In our research, only 2 participants provided quotes that could have been viewed as suggestive of this theme, but they were coded into “connection” and the “freedom of access” theme, as they talked of how they were able to access online support even at times of illness flare-up. Therefore, the SCENA model (Self-presentation, Connection, Exploration, Narration, and Adaptation) proposed by Merolli et al [39] to explain their results may be considered a valid model despite there being some differences, which can arguably be related to the different sample being examined and the fact that our work focused solely on online support groups rather than a range of social media.

The lack of evidence for the “adaptation” affordance in this research could simply be because it was not commented on by participants or because the affordance does not exist here. It is important to consider the differences in the “object” studied, as this research focused on online support groups rather than social media as studied by Merolli et al [39]. “Social media” encompasses a wider variety of platforms such as blogs, social networking sites, video-sharing sites, and chat rooms. This wider variety could afford different uses and outcomes than online support groups alone. This might too account for the different emergent themes found within each affordance compared with Merolli et al [39]. Another possible explanation for the differing findings is that different health conditions were examined. Users perceptions may therefore differ based on their goals, experiences, and beliefs, which is an important factor in affordance theory [38].

Outside of the health domain, researchers have attempted to further develop the concept of therapeutic affordances by categorizing them into types based on their underlying function [41-43]. The results of this study can be interpreted using the framework proposed by Zhao et al [43], in which the perceived affordances are categorized into physical, cognitive, affective, and control functions. “Connection” may be seen as an affective affordance, allowing users to find a community, seek support, and feel less alone. “Exploration” may best be categorized as a cognitive affordance as it helps to facilitate the user’s thinking and learning. “Self-presentation” may fall under control, as it allows users to control their environment, by maintaining their privacy for example. However, as Zhao et al [43] explain, affordances do not need to fall into these categories exclusively; for example, “narration” may be both affective, in providing reassurance or distress, and cognitive as it allows users to learn from others’ experiences.

These theoretical frameworks allow us to understand the user’s perceptions of and reactions to online support group use. By interpreting the results in relation to previous research in endometriosis, we can further improve our understanding of the affordances we have found. “Connection” was the most prominent therapeutic affordance in this study, with women frequently expressing their gratitude at finding others with endometriosis, who could understand their struggle and offer them support. Past research has reported that contact with other women with endometriosis is highly valued in this population [16,20]; however, it can be difficult to obtain [23]. This might explain the large number of responses relating to “connection,” as using online support groups can enable them to achieve such contact. Many women in our sample talked of how alone and isolated their condition made them feel, which has been a common theme in previous qualitative research [12,13,31]. The lack of an understanding support network further intensifies this isolation [13], and so finding those with experience and knowledge can be a great sense of support [20].

The “exploration” affordance also received significant attention in this study, and in particular, women talked of the ability to use online support groups to search for information related to their treatment. Previous literature has highlighted the struggles that women face with regard to their treatment, including lack of effective treatments [13], pain continuing post treatment [23], and concerns over side effects [44]. Furthermore, women have often felt let down by medical professionals, or complained of a lack of information from them [4,12,13,18], feelings which were also reiterated in this study. They have therefore taken it upon themselves to search for the information they need, and the Internet and online support groups provide a platform to do this [31]. Finally, research has indicated the tendency for women to want to self-manage their condition, and the empowering experiences of such [16,45], which again was a common theme in this research.

Comparisons with previous research suggest that the affordances enabled by online support groups, which were revealed in this research, may be effective aids when coping with and managing endometriosis. These results imply that the use of online support groups might form a meaningful part in the self-management of endometriosis. However, until clinicians can make decisions about whether to recommend their use for such, further research is needed that accurately measures the effect of support groups on health outcomes. Recently, Merolli et al [46] studied patient-reported outcomes (PROs) of social media use in those with chronic pain and found that it had a positive effect on psychological, social, and cognitive aspects of health. Similar research is needed to corroborate these findings in those with endometriosis before online support group use can be recommended as a tool for self-management.

It is also necessary to consider the negative outcomes that were reported in the research, such as feeling disheartened by others’ experiences. By accurately measuring PROs in future research, it might be possible to determine exactly how much of an effect these negative experiences are having on group users. It may also be useful in future research to try to measure the potential contribution of individual differences on perceived affordances. It may be the case that the therapeutic affordances offered by online support groups are only effective for certain types of individuals. Furthermore, our research cannot be generalized to other conditions, and so to further our understanding of the therapeutic affordances of online support group use, more research is needed to consider them in other chronic health conditions.

Our findings highlight a number of concerns that online support groups users may have that may affect the quality of their online experience. Our analyses revealed concerns over the accuracy and credibility of online information. One possible strategy to address these concerns might be to deploy an online moderator, such as a health professional, who can directly intervene to correct any misinformation or to validate information provided by a group member. Other “lay” moderators may similarly have a role to monitor online activity and ensure that members engage in a mutually respectful way. In terms of privacy concerns, a clearer statement of the privacy policy within online support groups may help to address this issue, although it should be noted that such concerns were restricted to Facebook groups. Finally, although there is clear value in people sharing their experiences, views, and opinions online, we did note that both negative and positive stories can have a detrimental effect on users. This is a difficult issue to address but may be dealt with partly through the development of specific subforums that focus on specific issues or experiences. In addition, there may be subforums devoted solely to “good news” and members would, we hope, have more freedom to choose the content they wish to be exposed to.

**Study limitations**

This study has a number of potential limitations. First, it is not possible to determine how representative this sample is of the whole population. Recruitment messages indicated that participants would be asked about their experiences of using such groups, and so there may have been a tendency for only those with more positive experiences to participate. Second, although the recruitment message was posted on 3 online support groups, more than half of respondents (62.3% 43/69) were recruited from 1 group, the one hosted by Facebook. The support groups used varied considerably, both by platform and by group purpose; for example, one focused more on fertility issues associated with endometriosis, whereas another provided users with access to specialists. Although the motives for accessing the groups were fairly standard across respondents, these differences may have enabled users with different opportunities and therefore different affordances. Some evidence for this was highlighted by the “self-presentation” theme, in which participants from the Facebook group expressed more concerns over the privacy of the group than those recruited from the other 2 groups. Unfortunately, as specific between-group comparisons were not made, it is not possible to understand the potential influence of group type on the resulting affordances. However, the inclusion of a variety of group types will, we hope, have provided a more diverse range of participant characteristics and experiences.

### Conclusion

This study has offered a unique exploration into the therapeutic affordances of online support group use in women with endometriosis. Through our analysis of the responses by participants to a series of open-ended questions, it has been possible to identify a number of therapeutic affordances arising from engagement with online support groups. Our findings reveal many positive aspects that may potentially benefit group members. However, several negative aspects are also revealed, particularly in relation to communication, personal information, and the presence of negative stories online. A number of strategies are recommended to mitigate these problems, including the presence of an explicit privacy policy, sections of the group where positive stories can be shared, and the presence of a health professional to address misleading or inaccurate information.

## Abbreviations

PRO: patient-reported outcome
